# An Overview of Vascular Dysfunction and Determinants: The Case of Children of African Ancestry

**DOI:** 10.3389/fped.2021.769589

**Published:** 2021-12-10

**Authors:** Edna N. Matjuda, Godwill Azeh Engwa, Constance R. Sewani-Rusike, Benedicta N. Nkeh-Chungag

**Affiliations:** ^1^Department of Human Biology, Faculty of Health Sciences, Walter Sisulu University PBX1, Mthatha, South Africa; ^2^Department of Biological and Environmental Sciences, Faculty of Natural Sciences, Walter Sisulu University PBX1, Mthatha, South Africa

**Keywords:** vascular dysfunction, obesity, cardiovascular risk factors, African children, vascular function

## Abstract

The balance between dilatory and constrictive factors is important as it keeps blood vessels in a homeostatic state. However, altered physiological processes as a result of obesity, hypertension, oxidative stress, and other cardiovascular risk factors may lead to vascular damage, causing an imbalance of vasoactive factors. Over time, the sustained imbalance of these vasoactive factors may lead to vascular dysfunction, which can be assessed by non-invasive methods, such as flow-mediated dilation, pulse wave velocity, flow-mediated slowing, retinal vessel analysis, peripheral vascular reactivity, and carotid intima-media thickness assessment. Although there is increasing prevalence of cardiovascular risk factors (obesity and hypertension) in children in sub-Saharan Africa, little is known about how this may affect vascular function. This review focuses on vasoactive factors implicated in vascular (dys)function, highlighting the determinants and consequences of vascular dysfunction. It further describes the non-invasive methods used for vascular (dys)function assessments and, last, describes the impact of cardiovascular risk factors on vascular dysfunction in children of African ancestry.

## Introduction

Cardiovascular diseases (CVDs) are a major cause of morbidity and mortality worldwide. In 2019, an estimated 17.9 million people died from CVDs, representing 32% of all global deaths (1). In sub-Saharan Africa (SSA), the disability-adjusted life years (DALYs) due to CVDs increased from 90.6 million in 1990 to 151.3 million in 2017 (2). CVDs in SSA are of major concern as they pose a challenge on an already strained health system (3). Although the prevalence of CVDs is higher in adults, the risk factors for CVDs, including obesity and hypertension, are increasing among children in SSA (4).

There is evidence that risk factors for CVDs, including obesity, hypertension, and hyperglycemia, begin early in life and may be associated with vascular dysfunction (5, 6). Also, it is reported that vascular dysfunction, an early initiator of CVD, begins in childhood and may lead to CVDs and associated complications in adulthood (7). Vascular dysfunction, which includes endothelial dysfunction, microvascular dysfunction, and stiffening of large arteries, results when the homeostatic function of relaxation and contraction of blood vessels is affected (8).

The endothelium is a major layer of blood vessels, and it is regulated by the release of potent vasodilators, such as nitric oxide (NO), prostaglandin I2, hydrogen sulfide, endothelium-derived hyperpolarizing factor as well as contracting factors, such as endothelin, prostacyclin, and thromboxane (9). A balance between vasodilatory and vasoconstrictive factors is important as it keeps blood vessels in a homeostatic state (10). Changes in the release of vasoactive factors, such as decreased bioavailability of NO, may lead to endothelial dysfunction. Endothelial dysfunction, along with other risk factors, such as aging, inflammation, obesity, increased salt intake, smoking, and alcohol consumption, could contribute to the development of arterial stiffness (8). Sustained arterial stiffening may predispose the intima layer of the affected blood vessels and may contribute to the development of atherosclerosis (11). Obesity is one of the major risk factors for the development of vascular dysfunction and CVDs (12). It increases the concentration of circulating free fatty acids and alters anti-inflammatory and pro-inflammatory cytokines that are released from visceral fat. These functional and structural changes affect the microvasculature, leading to vascular dysfunction and possibly CVDs (13). Also, oxidative stress is reported to affect vascular function as free radicals are shown to affect the availability of NO, leading to endothelial dysfunction (14). Free radicals can equally affect enzymes implicated in the regulation of the extracellular matrix of the blood vessel wall, leading to arterial stiffness (15).

It is reported that vascular dysfunction is central to the origin of CVDs (16). Moreover, there is increasing prevalence of cardiovascular risk factors, such as obesity and hypertension, in African children. A study conducted among adolescents in Fetakgomo Municipality, Limpopo Province of South Africa found that the prevalence of obesity was 35% (17). Another study carried out in the Eastern Cape Province of South Africa documented a 19.8% prevalence of obesity in children aged 6–9 years old (18). A recent meta-analysis study reports an increased prevalence of hypertension among African children aged 2–19 years (19). Although the prevalence of cardiovascular risk factors, such as obesity and hypertension, in children in SSA is on the rise, little is known about how these factors may affect vascular function. Hence, this review intends to give an overview of bioactive factors in the regulation of vascular function. It also discusses the causes of vascular dysfunction along with the methods used for assessment. It further highlights the key determinants of vascular dysfunction and the associated consequences and provides evidence of vascular dysfunction in children and adolescents of African ancestry.

### Vascular Function

**Figure 1:**
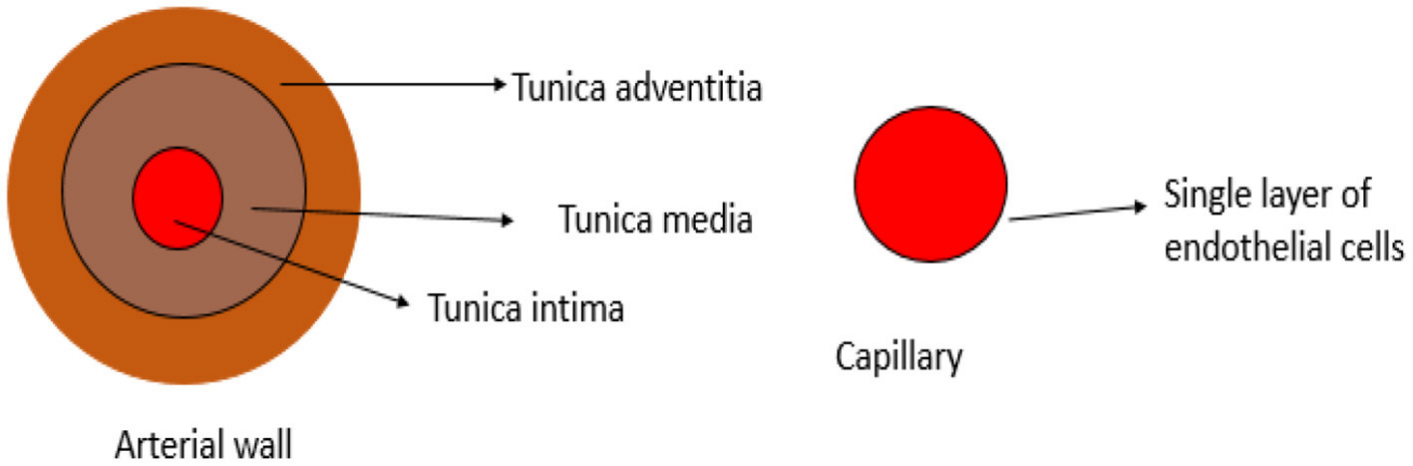
Cross-section of layers in the arterial wall.

The vascular system is made up of blood vessels, such as arteries, veins, and capillaries (20). Blood vessels are organized in hierarchal levels with complex and different configurations designed to ensure efficient exchange of nutrients and waste in and between tissues throughout the body. Large arteries with diameters above 6 mm transport oxygenated blood from the heart to smaller arteries ranging between 1 and 6 mm in diameter, then to the arteriolar network with diameters of 100–1,000 μm, and last into capillary beds of 10–15 μm in diameter (21). The arterial wall is an organized structure composed of matrix proteins (collagen fibers oriented in various directions and elastic lamellae), vascular smooth muscle cells (VSMCs), and other matrix components, such as glycosaminoglycans and endothelial cells in the inner layer (22). The cross-sectional layers of the arterial wall are shown in Figure 1. The endothelium is a thin monolayer of simple squamous cells lining the inner surface of the whole cardiovascular system (23, 24). It was once thought to be just an inert layer wrapping all endovascular surfaces. However, over the last four decades, research on the endothelium has become enormous, and its results have led to an understanding of its complex functions (25). It forms a biocompatible barrier between the circulating blood and all the underlying tissues (26). The endothelium plays an essential role in vascular function through several mechanisms, including the synthesis and release of substances that act in an autocrine and/or paracrine form. It controls all cardiovascular activities by releasing several vasoactive agents (27). The endothelium-derived dilating and contracting factors are balanced under physiological conditions so that vascular homeostasis is maintained in favor of vasodilation. Dilatory factors include NO, hydrogen sulfide, prostacyclin (prostaglandin I2), and endothelium-derived hyperpolarizing factor, whereas contracting factors include endothelin, thromboxane, and asymmetric dimethyl arginine **(**ADMA) (27). Microcirculation is the terminal vascular network of the systemic circulation comprising microvessels with a diameter of <20 μm. These microvessels consist of arterioles, postcapillary venules, and capillaries. Microcirculation is regarded as the last destination of the cardiovascular system and is ultimately accountable for the transfer of oxygen from the red blood cells in the capillaries to the parenchymal cells where oxygen is delivered to fulfill the energy requirements of the tissue cells (28). The capillaries consist of a single layer of endothelial cells (29). The distensibility and elasticity of arteries keep a relatively fixed blood pressure regardless of the pulsating nature of blood flow by each heartbeat. Arteries expand as a result of receiving blood expelled from the heart during systolic contraction and eject it to the periphery during diastole to supply the peripheral circulation with a steady flow of blood during systole and diastole cycles (30). Some of the major vasoactive factors implicated in the vascular function of blood vessels are discussed below.

### Vasoactive Factors

#### Thromboxane and Prostacyclin

Prostacyclin and thromboxane are vasoactive factors implicated in the regulation of blood vessel relaxation and contraction. Although prostacyclin also known as prostaglandin I2 is a vasodilator, thromboxane is a vasoconstrictor. Prostacyclin and thromboxane are produced from the endothelium of blood vessels (31). Prostaglandin H2 is produced following the enzymatic degradation of phospholipid membrane in the endothelium by phospholipase enzyme to release arachidonic acid (AA) (32). AA is then metabolized by cyclooxygenase-1 (COX-1) or cyclooxygenase-2 (COX-2) to produce prostaglandin H2, which is a precursor for thromboxane synthase, prostaglandin synthase, and prostacyclin synthase. Under physiological conditions, COX-1 is expressed in most tissues, whereas COX-2 is expressed by inflammatory cells, such as macrophages, and it leads to the production of thromboxane, which plays a role in platelet aggregation, vasoconstriction, and proliferation (33, 34).

The platelets remain in their inactive state as they circulate through the blood vessels of the intact endothelium. This inactivated state is sustained by continuous secretion of prostacyclin as well as the absence of pro-inflammatory factors that can activate COX-2. Once there is a break in the endothelium, platelets become activated by thromboxane, which initiates the aggregation of platelets into a growing thrombus through the activation of G-protein. This activates phospholipase C to hydrolyze phosphatidylinositol phosphate to diacylglycerol and inositol triphosphate as well as increases calcium ion accumulation to directly heighten VSMC contraction (35, 36). Following the release of prostacyclin, it acts on VSMCs through prostacyclin receptors linked to the activation of membrane-bound adenylate cyclase, which converts adenosine triphosphate (ATP) to cyclic adenosine monophosphate (cAMP). Accumulation of cAMP as a result of prostacyclin leads to vasodilation and inhibition of platelets aggregation (37).

#### NO

The most important vasoactive factor is NO as it plays a crucial role in the vasculature stimulating VSMC relaxation and, thus, controlling vascular resistance and blood pressure. It also eliminates free radicals and prevents build-up of plaque (38). As blood flows through the vessels, endothelial cells detect shear stress exerted by the pressure of blood and respond by releasing acetylcholine to act on its endothelial receptor, which triggers excessive release of calcium ions from the endogenous storage sites (39). The released calcium ions attach to calmodulin protein in the cytoplasm of the cell to form a calcium-calmodulin complex, which activates the endothelial nitric oxide synthase (eNOS). The active form of this enzyme catalyzes the conversion of L-arginine and oxygen to citrulline and NO molecule. There are three isoforms of mammalian NOS, namely, neuronal NOS (nNOS), inducible NOS (iNOS), and endothelial NOS (eNOS) of which the latter is the main source of NO in the endothelium (40). To apply its dilatory effects, NO diffuses to adjacent VSMCs, where it binds to the heme moiety of cytosolic guanylate cyclase (GC). This active enzyme, in turn, activates guanosine triphosphate to its active form, that is, cyclic guanosine monophosphate (cGMP) (41). It is the cGMP that facilitates the dephosphorylation of the myosin light chain, and this process induces the dissociation of myosin and actin filament resulting in VSMC relaxation (41).

#### Endothelin

Endothelin is a vasoconstrictor that exists in three isoforms, namely, endothelin-1 (ET-1), endothelin-2 (ET-2), and endothelin-3 (ET-3). Three different genes encode endothelin, which gives rise to three different precursors of pre-pro-endothelin (42). Pre-pro-endothelin-1 is the first product encoded by the ET-1 gene (43). This precursor is transformed into pro-ET-1 by removal of a short sequence by a signal peptidase. The pro-ET-1 is then converted to big ET-1 through the activity of furin, a maturing enzyme. Mature ET-1 is obtained by proteolytic cleavage of big ET-1 by endothelin converting enzyme into a small active 21 residue ET-1 (44). Once ET-1 is formed and released from the endothelium, it acts through two types of receptors, namely, endothelin A (ETA) and endothelin B (ETB) receptors. Currently, ET-1 and ET-2 are known to have the strongest affinity for both receptors, whereas ET-3 binds only on ETB (42). ET-1 binds to these receptors on the VSMCs. ETA and ETB are coupled to G-protein to form inositol triphosphate (IP3). This IP3 accumulates in the sarcoplasmic reticulum, leading to the secretion of calcium ions, which, in turn, results in the contraction of VSMCs (45). It is documented that ET-1 is the most potent vasoconstrictor. Moreover, ET-1 is suggested to decrease endothelium-dependent vasodilation. This may be due to the combined effect of ET-1–induced vasoconstriction and, to a lesser extent, ET-1–mediated inhibition of NO production, which together affect the balance between dilatory and constrictive factors in favor of the latter (46). The normal vascular endothelium is considered as a gatekeeper of cardiovascular health, whereas harmful stimuli, such as oxidative stress and inflammation, alter the normal endothelium function, leading to the development of vascular dysfunction (45).

#### ADMA

Dimethyl arginines are formed during the methylation of L-arginine residues within specific proteins, a process that is catalyzed by arginine methyltransferase. ADMA is released following a cleavage of methylated proteins during physiological protein turnover (47). Under physiological conditions, ADMA is excreted in urine. However, under pathological conditions, its elimination may be blocked due to hypertension, hypercholesterolemia, diabetes mellitus, and chronic kidney failure (48). As such, there is increased ADMA concentrations in the circulation, which, in turn, competes with L-arginine for the NOS binding site, thereby inhibiting the production of NO (49). Furthermore, both ADMA and L-arginine are transported into the cell through a cationic amino acid transporter; therefore, they compete with each other at the transporter to enter the cell where they are being catalyzed by NOS. As such, the production of NO depends on the balance between L-arginine and ADMA because they both compete for NOS and cell transport (50).

#### Endothelium-Derived Hyperpolarizing Factor

Endothelial-derived hyperpolarizing factor (EDHF) plays an important role in controlling the vascular tone in the microvasculature (51). Whereas blood vessel relaxation is easily impaired as a result of decrease in NO, EDHF activity of relaxation is enhanced to preserve the homeostasis of blood vessels. This activity of EDHF induces the formation of a disulfide bond between two cysteine 42 residues of each of the adjacent chains in protein kinase G (PKG) (51). This leads to the opening of large Ca^2+^-dependent channels, resulting in hyperpolarizing and vasodilation (52). The vasoactive factors and their functions are summarized in Table 1.

**Table 1:** Vasoactive factors and their functions.

**Vasoactive factors**	**Functions**	**Citation**
Endothelium-derived hyperpolarizing factor	Vascular relaxation in the microvascular beds	(52)
Nitric oxide	Stimulates vascular smooth muscle relaxation, modulate vascular tone and, controls blood pressure	(38)
Thromboxane	Powerful vasoconstrictor and stimulate platelet aggregation	(35)
Prostacyclin	Inhibit platelet aggregation and is a potent vasodilator	(36)
Endothelin	Potent vasoconstrictor and counteracts nitric oxide	(45)
Asymmetric dimethyl arginine	Inhibitor of nitric oxide synthesis	(49)

### Vascular Dysfunction

Vascular dysfunction comprises dysfunction of the endothelium (endothelial dysfunction), microvascular dysfunction, and large artery dysfunction due to arterial stiffness (9). Endothelial dysfunction is characterized by an imbalance between constrictive factors and dilatory factors, increased concentration of reactive oxygen species (ROS), pro-inflammatory factors, and decreased NO bioavailability (41). The production of NO depends on its precursor, L-arginine, which is synthesized in healthy humans from l-citrulline by endogenous synthesis. This means that reduced levels of L-arginine and l-citrulline contribute to NO insufficiency. Also, free radicals, such as superoxide (O_2_^·^), may react with NO to form peroxynitrite (ONOO^−^) radicals, thereby reducing NO levels (40). A variety of ROS-producing systems, such as NADPH oxidase, xanthine oxidase, eNOS, and enzymes of the mitochondrial respiratory chain, are found within the vascular wall. Moderate levels of ROS have important signaling roles under physiological conditions. Excessive and persistent production of ROS, however, when exceeding the present antioxidant defense enzymes, leads to oxidative stress and decreased NO production (53). It is documented that NO production can also be decreased by ADMA, which competes with the substrate of eNOS, L-arginine, thus inhibiting NO production (54). Endothelial NO is one of the major dilatory factors, and its insufficiency contributes to elevated vascular constriction (55). A study documents that deterioration of NO results in increased levels of ET-1, which is a major vasoconstrictor, leading to a decrease in endothelial dilatory capacity (56). A study conducted in South Africa finds that ADMA is inversely correlated with carotid intima-media thickness (57). Another study documents that black men and women have higher central systolic blood pressure, higher plasma ADMA, and lower urinary nitrate than their white counterparts. This suggests potential increased chances for vascular damage and large arterial stiffness in people of African ancestry in the future as a result of endothelial dysfunction (58).

Microvascular dysfunction is a condition characterized by impaired endothelium-dependent dilation of isolated arterioles. It is documented that microvascular dysfunction precedes and predicts the development of conduit artery atherosclerosis and its determinants (59). Abnormal microvascular function may occur as a result of structural alterations in small arteries due to inward eutrophic remodeling without overall growth of the cell, leading to decreased vasodilator reserves and changes in distensibility of arterioles (60). A study reports that remodeling (damage) of the small artery plays a crucial role in the increase of vascular resistance. This damage in the small arteries, characterized by the thickening of the carotid intima, may be considered as the first manifestation of target organ damage before it occurs in the large arteries (61). More direct impairment of microvascular function occurs as a result of persistent ischemia, manifesting as reduced maximal flow on computerized tomography without the presence of conduit stenosis (59). Microvascular dysfunction is linked to several conditions, such as smoking, obesity, hypertension, and diabetes (62). As such, microcirculatory alteration noted in the renal and retinal systems are extensively studied to investigate the predictive role of glycemic variations early in diabetes (60).

The loss of arterial elasticity, also called arterial stiffness, describes the mechanical property of artery resistance to deformation (63). The stability, compliance, and resilience of the vascular wall are dependent on the activity of two major scaffolding proteins, namely, elastin and collagen (64). The content of these proteins is usually made stable by a dynamic but slow process of their synthesis and degradation. Dysregulation of this balance between their production and degradation commonly stimulated by inflammatory molecules leads to the overproduction of collagen at abnormal levels, which diminishes the normal elastin content. This affects the elasticity and resistance of the arteries, contributing to vascular stiffness (63). With every heartbeat, a pulse wave generated by the arteries travels through the vascular bed until it reaches peripheral resistance or any bifurcation point, producing a new reflected wave back to the heart (65, 66). The reflected wave velocity and the stage of the cardiac cycle in which it happens (during systole or diastole) depends on the peripheral vascular resistance, elasticity primarily of the large arteries, and central blood pressure (66). In healthy individuals, arteries are compliant, and therefore, the reflected wave is slow and returns to the heart during the diastole cycle. However, in individuals with arterial stiffness, the reflected wave reaches the heart early during systole cycle. As a result, this increases the systolic blood pressure with a subsequent increase in cardiac workload to overcome the augmented systolic blood pressure (30, 66).

### Assessment of Vascular Function

Vascular function constitutes endothelial function and functioning of the microcirculation and macrocirculation. Endothelial function is mostly assessed by flow mediated dilation (FMD) techniques, which require occlusion. Retinal imaging is mostly used to assess the functioning microcirculation, and the macrocirculation function can be assessed by measuring the pulse wave velocity (PWV) as discussed below (67, 68).

#### FMD

Vascular function can be assessed by numerous methods, including invasive and non-invasive techniques (69). Among the non-invasive techniques, FMD is one of the validated methods for the assessment of vascular function. The method involves ultrasound imaging in stages, at baseline (before occlusion) and during reactive hyperemia (5 min after occlusion of the artery) (70). Endothelial cells lining the artery sense an increase in blood flow and react by generating NO, which causes the diameter of an artery to increase to accommodate the increased demand (71). Such a response is known as FMD. In this technique, a blood pressure cuff is inflated in the forearm to temporarily occlude the brachial artery for a few minutes. This is followed by deflation of the pressure cuff to restore blood flow to the forearm and using an ultrasound to measure the increased diameter of the brachial artery caused by the sudden increase in blood flow (69, 71).

Impaired FMD is linked with conditions predisposing CVDs and is known to be the earliest step in developing subclinical target organ damage (72). In addition, assessment of FMD can classify individuals at low, moderate, or high risk for future clinical events (69). FMD provides valuable prognostic data and is considered the gold standard for assessing endothelial dysfunction (72). However, it has a few limitations that are worth consideration. First, the absence of standardization and differences in placement or positioning of the cuff/probe makes comparison of results difficult. Results may be operator-dependent as the technique requires expertise in the placement of the probe on the arm to identify the pulsating artery. Moreover, changes in structure of the arteries and impaired dilation may be a limiting factor during an FMD test (69).

#### Flow-Mediated Slowing

Flow-mediated slowing (FMS) can be described as the minimum PWV during reactive hyperemia representing endothelial function (73). A vicorder device is used to perform this test, in which the participant is requested to rest in a supine position for at least 20 min before oscillometric cuffs are wrapped around the upper arm and wrist. FMS assessment commences with baseline measurement of PWV for 4 min followed by 5 min of blood pressure occlusion and finally, 4 min of a postocclusion in which the pressure cuff is released (74). At the end of the test, minimum PWV (m/s) during hyperemia is recorded. PWV is calculated by dividing the arterial length by transit time between the upper arm and wrist. Particularly, the length is measured directly using the device to bypass body contours between the two midpoints of the two cuffs (73). FMS is easier to perform than FMD and is less operator-dependent. As a result, some studies report that FMS seems to be a promising and feasible method for endothelial function assessments (75, 76).

#### Peripheral Vascular Reactivity Assessment

Endothelial dysfunction can also be measured non-invasively by using a quantitative magnetic resonance imaging (MRI) technique that measures the peripheral vascular reactivity in the superficial femoral artery and vein (77). In this method, participants are required to lie in a supine position on the imager table whereby an eight-channel extremity transmitter–receiver coil is used for assessment. Following 2 min of a baseline period, a sphygmomanometer cuff is applied to the upper right thigh proximal to the targeted vessels, and then it is quickly inflated with a pneumatic pump for a 5-min occlusion period to the target pressure of 220 mmHg. This is followed by a post-occlusion period of 5 min (78). Vessel-wall imaging is done at baseline, occlusion, and post-occlusion to quantify superficial femoral artery luminal flow-mediated dilation, venous oxygen saturation, and arterial blood flow velocity (78). A study reports that methods of quantitative MRI can detect endothelial dysfunction in the presence of overt cardiovascular disease. However, so far, the use of this instrument is limited to research to identify biomarkers for disease progression (77).

#### Retinal Microvasculature Assessment

The retina is rich with blood vessels and, thus, shares similar anatomical features and physiological properties with blood vessels in the body. As such, visualization of the retinal vasculature allows direct non-invasive assessment of the microvasculature in relation to health and diseases of the vascular system (79). Retinal microvascular changes, such as arteriolar narrowing, arteriovenous nicking, focal arteriolar narrowing, and changes in static retinal vascular caliber, are reported to be early signs of hypertensive retinopathy and atherosclerosis (80). Analysis of the retinal image is of importance as it assists in early diagnosis of diabetic and hypertensive retinopathy and CVDs (80). A portable and easily movable fundus camera is a tool used to assess changes in the retina, retinal vasculature, and macula of the eye using a low-power intricate microscope in a cost-efficient manner (80, 81). Furthermore, dynamic measurements, such as maximal retina vessel dilation, can also be used to further assess retinal microcirculation (77). The digital interior imaging of the eye through a fundus camera has sensors that convert a light signal into an electric signal, and the result is stored in the form of a pixel (80). Static digital photographs of the retina are taken from both eyes, and computer-based software is used to measure the diameter of arterioles and venules (79). The diameter of the central retinal artery (CRAE) and central renal vein equivalent (CRVE) are calculated. Also, other structural changes, including arteriovenous nicking (AVN) and focal arteriolar narrowing (FAN), are assessed (79). To perform this test, the patient is required to sit in front of the camera with the patient's forehead against the bar. The trainer focuses and aligns the fundus camera on the pupil, and the shutter button is released, thus, firing a flash that forms a photograph of the interior surface of the eye (82). A fundus camera can assist health workers to control vascular diseases affecting both the central and peripheral retina, and it can help patients understand the extent of their cardiovascular health condition (82). An observational study among 40- to 60-year-old adults in the United Kingdom shows that retinal fundus imaging alone may predict multiple cardiovascular risk factors, such as age, gender, and systolic blood pressure (83).

#### Pulse Wave Velocity

At the end of the ventricular ejection phase, a pressure wave generated from the heart propagates along the arterial tree (69). PWV is defined as a measure of the speed of the arterial pressure wave traveling from the heart along the aorta to the large arteries. It is calculated as the distance of the pressure wave between the arteries/transit time. PWV is the most widely used measure for arterial stiffness (84). There are different types of PWV measurements with carotid-femoral PWV (cfPWV) and brachial-ankle (baPWV) being the most commonly used methods in clinical settings and research (84). PWV can be assessed non-invasively using a vicorder device, and it is referred to as the “gold standard” measurement for arterial stiffness because it is a reliable, inexpensive, and simple non-invasive tool to identify or detect CVD risk in its earliest stages (84). A study finds that the 10th, 50th, and 90th percentiles of cfPWV assessed using a vicorder were, respectively, 4.8, 5.57, and 6.6 m/s as reference values for adolescents aged 18 years old (85).

Apart from the vicorder, the sphygmocor cardiovascular management suite (CvMS) has been used in the field as a non-invasive method for PWV and aortic pressure waveform assessment. This device depends on applanation tonometry to detect radial, carotid, and femoral blood pressure waveforms (86). Studies utilize this device to measure PWV (87, 88). A study in South Africa has equally utilized this device to assess PWV in pre-eclamptic women (89). Although this device is reported to be effective in assessing PWV, its major disadvantage is difficulty in obtaining the peripheral waveform. Also, the device is technically difficult to use, and it is operator-dependent in identifying the peripheral signal (86, 90).

Recently, a new device called the Sphygmocor XCEL, which makes use of the volumetric displacement (cuff-based) technique to obtain pulse information, was developed (86). It is used to measure arterial stiffness and wave reflection strength (91). A study in South Africa reports that further studies are required to investigate the accuracy of PWV measurements by Sphygmocor XCEL (89). This device is preferable over the Sphygmocor CvMS because it is not operator-dependent (92). Furthermore, there is no need for an electrocardiogram to be aligned sequentially to acquire signals when assessing cfPWV using Sphygmor XCEL. However, Sphygmocor CvMS is more suitable in research than Sphygmocor XCEL in measuring high-frequency components of the waveform (86).

Another device for the measurement of PWV and central systolic blood pressure is the Complior. This device measures the PWV between the carotid and radial arteries using piezoelectric clips (sensors) placed around the neck and the wrist (93). This device is suggested to be accurate and reliable in the non-invasive assessment of PWV and is utilized in studies in South Africa to measure PWV (94–97). However, one of the limitations of this device is that it is operator-dependent in accurately positioning the sensors in the various arteries to measure the waveform. This may lead to discrepancies between the distance measured between the sensors and the actual path length traveled by the pulse wave. Furthermore, the sensors are highly sensitive to motion and may be affected by the positioning of the arteries (94, 98).

#### Carotid Intima-Media Thickness Assessment

Carotid intima-media thickness (cIMT) is the thickness of the intimal and medial layers of the carotid arterial wall, and it can be measured non-invasively using a scanner imaging device (99). The test is performed using a sonography with a high frequency of 7.5 MHz linear array transducer. The patient is required to lie in a supine position, and the common carotid artery is visualized at 1 cm proximal to its bifurcation (100). The cIMT is described as the length between the leading edge of the luminal echo to the leading edge of the adventitia of the media (101). It is documented that cIMT >0.9 mm is denoted as a marker of asymptomatic organ damage. Moreover, intima media thickness (IMT) is accepted as an earliest marker of atherosclerotic vascular disease, and screening of IMT can help physicians to classify patients with cardiovascular risk into lower or higher risk categories (102). A study conducted in South Africa reveals that cIMT is elevated in females with HIV aged 35–45 years old in Elandsdoorn, Limpopo (103). A study among a group of individuals from Johannesburg and Limpopo, South Africa, finds that increased cIMT is associated with cholesterol (104). In the North West Province of South Africa, lower cIMT was associated with physical activity among female teachers (105).

### Determinants of Endothelial Dysfunction

It is known that risk factors for CVDs begin early in life (5, 6). A study finds that carotid bifurcation regions depicted widespread intimal lipid accumulation among newborn cadavers (106). Moreover, bifurcation anatomy affects blood flow, which causes endothelial injury (106). This indicates that endothelial dysfunction begins early in life. A study confirms that offspring have a distinct endothelial regulatory micro RNA profile at birth, which is associated with altered endothelial cell behavior during the first 3 months of life (107). It is documented that maternal total cholesterol (TC) concentrations increase in human pregnancy to meet the demands of the growing fetus (108). In some pregnancies, however, TC increases excessively mainly due to low-density lipoprotein cholesterol levels, a condition called maternal supraphysiological hypercholesterolemia in pregnancy, which is associated with endothelial dysfunction of the umbilical vein and early development of atherosclerosis in the fetal aorta (109). Furthermore, endothelial dysfunction is associated with various obstetrical syndromes, such as fetal growth restriction (FGR) (110). Evidence shows that FGR fetuses alter their cardiovascular function *in utero* to adjust to persisting suboptimal conditions, mainly chronic hypoxia (111). Changes in cardiovascular function secondary to utero-placental deficiency may result in permanent alterations in vascular structure (112). Fetal growth restriction leads to low birth weight. Children born with low birth weight experience catch up growth during their first years of life, thus, accumulating greater visceral adiposity, exposing them to an adverse metabolic outcome (110). All these findings suggest that maternal cardiovascular risk factors may affect the vascular function of the fetus and neonates.

Obesity, a multifactorial condition characterized by excess adipose tissue is a major determinant of vascular dysfunction and constitutes a serious worldwide health problem (113). The adipose tissue, where fat is stored in the body, is a type of connective tissue comprising lipid-filled cells (adipocytes) surrounded by a matrix of collagen fibers, blood vessels, immune cells, and fibroblasts. It consists of several cells with adipocytes being the most abundant. Other cells include stromal vascular fraction (SVF), endothelial cells, macrophages, stem cells, fibroblasts, and lymphocytes (114). Persistent accumulation of fat in the adipose tissue leads to adipocyte hypertrophy and hyperplasia (113). Adipose tissue hypertrophy (adipocyte cell size increases) and hyperplasia (increase in adipocyte number) occurs in childhood (115). The expansion of adipocytes leads to an increased release of free fatty acids and necrotic cell death due to hypoxia and inflammation (116). During physiological conditions, inflammation is regarded as a protective mechanism. However, obesity is accompanied by some degree of inflammation called low-grade inflammation (117) whereby the adipose tissue secretes high levels of pro-inflammatory adipocytokines, including tumor necrosis factor alpha (TNF-α), interleukin-6 (IL-6), resistin, and leptin, due to cell death by necrosis following hypoxia (113). This causes an infiltration of neutrophils, eosinophils, monocytes, and lymphocytes to clean up the dead cells (117). The resident macrophages in the adipose tissue release chemo-attractants for macrophages, which results in the persistent nature of chronic inflammation. This, in turn, promotes the inhibition of the production of adiponectin, an anti-inflammatory adipokine (117). Adiponectin is regarded as a beneficial adipokine in relation to metabolism with plasma concentration indirectly associated with trunk obesity, type 2 diabetes risk, and insulin resistance, whereas leptin positively correlates with waist circumference and is associated with the onset of insulin resistance (95, 118). TNF-α is known to trigger insulin resistance in obese individuals. IL-6 is known to be implicated in the pathways of insulin sensitivity, lipoprotein lipase downregulation and triglyceride synthesis (119). Persistent release of these pro-inflammatory markers, such as TNF-α and IL-6 results in decreased production of adiponectin (120). Decreased plasma levels of adiponectin promote the synthesis of arginase, a metalloprotease that catalyzes the conversion of L-arginine to L-orthinine and urea. The increased concentrations of arginase compete with eNOS for the substrate L-arginine. Increased arginase activity uncouples eNOS for the synthesis of NO, thereby leading to reduced production of NO (121). A decreased bioavailability of NO leads to endothelial dysfunction. Defect in the synthesis of NO can also be caused by high concentrations of ADMA in the plasma (122). ADMA is an endogenous competitive inhibitor of L-arginine for all three isoforms of NOS. High levels of ADMA block the synthesis of NO and limit the cellular uptake of L-arginine, thereby further disrupting the production of NO. In this manner, ADMA further affects the endothelial function (123).

Secreted inflammatory molecules, including pro-inflammatory cytokines, contribute to the generation of ROS (124). Since adipose tissue are known to secrete pro-inflammatory cytokines, they may promote the generation of ROS. As such, adipose tissue is regarded as an independent factor for the development of oxidative stress (125). ROS are highly reactive radicals derived from molecular oxygen, such as O^2−^, hydrogen peroxide (H_2_O_2_), hydroxyl radical (OH·), and ONOO^−^, that impair structural conformation of protein, DNA, and RNA in the cell, resulting in cellular dysfunction and cell death (126). Under physiological conditions, ROS contribute to cellular growth regulation, differentiation, and apoptosis (114). Furthermore, they are produced from endothelial cells by several enzymes, including NADPH oxidases, xanthine oxidoreductase (XOR), and mitochondrial enzymes, among many other sources (127). It is known that H_2_O_2_ has vasodilatory effects, whereas O^2−^ is a vasoconstrictor and leads to endothelial dysfunction (128). High levels of O^2−^ may react with NO to form an unstable free radical called ONOO^−^ (129). Furthermore, ROS can be produced from the uncoupling of eNOS (129). eNOS uncoupling may occur due to limited availability of the substrate L-arginine (128). As a result, eNOS may produce O^2−^ instead of NO, leading to more defect in the synthesis of NO and, hence, endothelial dysfunction (129). Also, small, dense, low-density lipoprotein (LDL) in the lumen is deposited into the subendothelial space where it becomes oxidized by ROS to become ox-LDL, which activates endothelial cells, causing expressed receptors for white blood cells on the surface (130). It is reported that ox-LDL induces the expression of ICAM-1 and VCAM-1, increasing the adhesive properties of the endothelium. The production of NO by endothelial cells is inhibited by ox-LDL. It is documented that ox-LDL leads to oxidative stress, producing high amounts of O^2−^, which inactivates NO to form ONOO^−^ (131). The decrease in NO as a result of ox-LDL leads to endothelial dysfunction.

Although hypertension is generally known be a consequence of endothelial dysfunction (132, 133), recent data suggest that hypertension may be a cause of endothelial dysfunction. There are reports that hypertension-induced endothelial dysfunction may be a result of hypertension-induced oxidative and inflammation (134). Hypertension-associated oxidative stress regulated by nicotinamide adenine dinucleotide phosphate (NADPH) oxidase and mitochondria show reductions in endothelium-dependent vasodilation to acetylcholine in carotid arteries of mice exposed to increasing intraluminal pressure as a result of increase in NADPH oxidase activity and vascular O^2−^ production (135). Also, obese hypertensive rats with perivascular inflammation show impaired endothelial function (136). Further, the activation of the innate immunity complement pathway, which regulates inflammation, is negatively associated with vascular endothelial function in hypertensives (137). All these studies support the notion that hypertension may be the cause of endothelial dysfunction.

### Consequences of Vascular Dysfunction

Endothelial dysfunction is a crucial risk factor for the development of high blood pressure as it not only impairs the control of the vascular tonus, but also alters structural function, such as the tunica intima of blood vessels (138). LDL as a result of hyperlipidemia, which is associated with obesity, may be deposited into the intima of blood vessels where they may be oxidized by ROS. This oxidized LDL (ox-LDL) activates the endothelial cells to induce monocyte recruitment into the endothelial wall (139). The recruited monocytes differentiate into macrophages that take up the ox-LDL via scavenger factors, resulting in intracellular lipid accumulation and subsequently the formation of foam cells (139, 140). Foam cells produce growth factors that cause the synthesis of collagen and VSMC to migrate into the intima, which begins to proliferate and secrete extracellular matrix, resulting in thickening of the arterial intima. Thickening of the intima can lead to severe CVDs, such as stroke, ischemic disease, and congestive heart failure later in life (139, 141).

It is known that early endothelial dysfunction decreases vascular relaxation and causes the infiltration of inflammatory cells, leading to mild inflammation in blood vessels (142). eNOS is formed in high concentrations in endothelial cells, specifically in the renal medulla, where it maintains medullary blood flow in response to renal vasoconstrictors, such as angiotensin II. Impaired activity of eNOS may be due to endothelial damage or extrinsic free radical activity altering NO activity (143). ROS may influence the effects of dilatory and constrictive factors, thus leading to elevated vascular resistance and acute kidney injury (144).

Sustained damage by hyperglycemia or other factors, such as hypertension in the microvessels of the retina results in diabetic retinopathy (145). Diabetic retinopathy is the main cause of blindness in high- and middle-income countries (109). Hyperglycemia increases hypoxia induced factor 1 (HIF-1) and insulin-like growth factor-1 (IGF-1). The overexpression of HIF-1 and IGF-1 and other factors activate Müller cells to transform into chronic inflammatory cells. Moreover, this induces overexpression and buildup of vascular endothelial growth factor (VEGF) causing fibroblast growth, thereby initiating fibrosis (146). VEGF is documented to stimulate angiogenesis and neovascularization, which are involved in the pathogenesis of proliferative retinopathy (145). Microvascular dysfunction can also result from arterial stiffness (147). Arterial stiffness is associated with normal and accelerated aging (147). The consequence of arterial stiffness includes augmented systolic blood pressure, which is characterized by pulse pressure (30, 148). Greater pulsatile pressure increases the pulsatile flow to penetrate deeper into the periphery and damage the microvasculature specifically in the brain and kidney (30).

### Vascular Dysfunction in Children and Adolescents of African Ancestry

The increasing prevalence of cardiovascular risk factors, such as hypertension, in SSA children has implications on their vascular health (4). However, very few studies assess the vascular function of children of African ancestry. A study in Kwa-Zulu Natal Province of South Africa shows that age and resting heart rate were positively associated with arterial stiffness among children aged 10–13 years old (149). Age could play an important role when assessing arterial stiffness (150). However, for a deeper understanding, it should be examined in conjunction with growth and maturation, given that body height at the transition from childhood to adolescence is documented to affect arterial stiffness. An association between resting heart rate and arterial stiffness in children is still lacking in the literature (149). A study conducted in the Eastern Cape Province, South Africa, among 6- to 9-year-old children finds that blood pressure parameters, such as mean arterial and diastolic blood pressure, increased with increasing PWV (151). This suggests that hypertension may result in vascular impairment in children. Another study conducted in Potchefstroom, North West Province of South Africa, in 6- to 8-year-old boys shows that oxidative stress is positively associated with cfPWV and carotid dorsalis pedis PWV in boys exposed to maternal cardiovascular risk compared with the non-maternal risk group (152). This suggests that oxidative stress may be an early mediator of vascular changes in children exposed to maternal cardiovascular risk. PWV significantly correlates with ADMA and systolic blood pressure (SBP) in a study conducted among 13- to 16-year-old children in the Eastern Cape Province of South Africa, suggesting that ADMA might be considered as a major risk factor of vascular dysfunction in adolescents (153). The PWV increased with cumulative time on ART in children living with HIV among primary school children in Cape Town, Western Cape Province of South Africa (154). In Mozambique, a study conducted among children with perinality-acquired HIV finds that PWV is higher in participants with increased visceral fat, elevated lipids, and insulin resistance (155). A study carried out in Egypt among 74 obese children aged 6–18 years finds a significant positive correlation between cIMT and BMI. cIMT equally shows a significant positive correlation with triglycerides and TC (156). Another study conducted in Egypt among 5- to 14-year-old children finds that cIMT is higher in obese children as compared with non-obese children. Further, obese children with elevated LDL and TC show increased risk for endothelial dysfunction and early signs of atherosclerosis (157). Thus, higher cIMT in obese children denotes increased risk for early vascular dysfunction. Exposure to risk factors of CVDs, such as hypertension and hyperlipidemia in obese children may induce alterations in the arteries, thus contributing to impaired endothelial function (156, 157). Higher PWV (carotid-radial, carotid-femoral, and carotid-dorsalis), diastolic blood pressure, and cIMT are reported in black boys than in white boys aged 6–8 years old in Potchefstroom, North West Province of South Africa. Moreover, black boys had increased levels of pentosidine, which is a biomarker for microvascular complications. However, arterial stiffness was not associated with pentosidine in both groups of boys, suggesting that vascular aging begins early in black population (158). Risk factors associated with vascular dysfunction in African children are summarized in Table 2.

**Table 2:** Vascular dysfunction and their associated risk factors in African children.

**Age**	**Number of children**	**Country**	**Type of study**	**Measure of vascular function**	**Outcome**	**Citation**
10–13	59	South Africa	Cross-sectional	PWV	Arterial stiffness was associated with age in boys.	(149)
6–9	303	South Africa	Cross-sectional	PWV	PWV increased with an increase in arterial pressure	(151)
6–18	74	Egypt	Cross-sectional	cIMT	cIMT correlated with BMI	(156)
13–16	244	South Africa	Cross-sectional	PWV and ADMA	PWV significantly correlated with ADMA	(153)
5–14	82	Egypt	Cross-sectional	cIMT	Increased cIMT in obese children	(157)
6–8	81	South Africa	Cross-sectional	PWV	High PWV observed in black boys as compared to their white counterparts	(158)
6–8	81	South Africa	Cross-sectional	PWV	Lipid peroxidation correlated with cfPWV	(152)
6–12	77	Mozambique	Cross-sectional	PWV	PWV higher in children with increased visceral fat, insulin resistance and increased lipids	(155)

*ADMA, Assymetric Dimethyl arginine; PWV, Pulse wave velocity; CIMT, Carotid intima-media thickness; BMI, Body mass index; cfPWV, Carotid-femur PWV*.

## Conclusion

Cardiovascular risk factors, such as obesity and hypertension, are known to be major contributors to the development of vascular dysfunction in children of African ancestry. Parameters of vascular function, such as PWV, cIMT, and ADMA, are used to assess cardiovascular risk in children of African ancestry. The presence of vascular dysfunction triggered by obesity, hypertension, oxidative stress, and inflammation in these children suggest a future risk of CVDs, such as stroke and heart attack in adulthood. However, only a few studies assess vascular changes in children of African ancestry, and such assessments are mostly limited to arterial stiffness and cIMT, as non-invasive methods along with a few vasoactive factors. Moreover, limited or no studies utilize FMD, FMS, retinal vascular assessments, and other recent PWV techniques to assess vascular function. These findings are, therefore, not sufficient to clearly describe the state of vascular dysfunction in children of African ancestry, and thus, additional studies with more robust methods for the assessment of vascular function, such as FMD and retinal microvasculature measurements are needed to provide sufficient information on vascular function in children of African ancestry and its implication.

## Author Contributions

GE and BN-C were involved in the development and conceptualization of this review. EM developed the literature with the assistance from GE, BN-C, and CS-R. All authors mentioned contributed to the final manuscript.

## Conflict of Interest

The authors declare that the research was conducted in the absence of any commercial or financial relationships that could be construed as a potential conflict of interest.

## Publisher's Note

All claims expressed in this article are solely those of the authors and do not necessarily represent those of their affiliated organizations, or those of the publisher, the editors and the reviewers. Any product that may be evaluated in this article, or claim that may be made by its manufacturer, is not guaranteed or endorsed by the publisher.
